# Host genetics and gut microbiota jointly regulate blood biochemical indicators in chickens

**DOI:** 10.1007/s00253-023-12814-8

**Published:** 2023-10-04

**Authors:** Xinwei Jiang, Boxuan Zhang, Fangren Lan, Conghao Zhong, Jiaming Jin, Xiaochang Li, Qianqian Zhou, Junying Li, Ning Yang, Chaoliang Wen, Congjiao Sun

**Affiliations:** Department of Animal Genetics and Breeding, College of Animal Science and Technology, China Agricultural University, Beijing, 100193 China

**Keywords:** Chicken, Blood biochemical indicators, Host genetic variations, Gut microbiota, Joint regulation

## Abstract

**Abstract:**

Blood biochemical indicators play a crucial role in assessing an individual’s overall health status and metabolic function. In this study, we measured five blood biochemical indicators, including total cholesterol (CHOL), low-density lipoprotein cholesterol (LDL-CH), triglycerides (TG), high-density lipoprotein cholesterol (HDL-CH), and blood glucose (BG), as well as 19 growth traits of 206 male chickens. By integrating host whole-genome information and 16S rRNA sequencing of the duodenum, jejunum, ileum, cecum, and feces microbiota, we assessed the contributions of host genetics and gut microbiota to blood biochemical indicators and their interrelationships. Our results demonstrated significant negative phenotypic and genetic correlations (*r* =  − 0.20 ~  − 0.67) between CHOL and LDL-CH with growth traits such as body weight, abdominal fat content, muscle content, and shin circumference. The results of heritability and microbiability indicated that blood biochemical indicators were jointly regulated by host genetics and gut microbiota. Notably, the heritability of HDL-CH was estimated to be 0.24, while the jejunal microbiability for BG and TG reached 0.45 and 0.23. Furthermore, by conducting genome-wide association study (GWAS) with the single-nucleotide polymorphism (SNPs), insertion/deletion (indels), and structural variation (SV), we identified RAP2C, member of the RAS oncogene family (*RAP2C)*, dedicator of cytokinesis 11 (*DOCK11*), neurotensin (*NTS*) and BOP1 ribosomal biogenesis factor (*BOP1*) as regulators of HDL-CH, and glycerophosphodiester phosphodiesterase domain containing 5 (*GDPD5*), dihydrodiol dehydrogenase (*DHDH*), and potassium voltage-gated channel interacting protein 1 (*KCNIP1*) as candidate genes of BG. Moreover, our findings suggest that cecal *RF39* and *Clostridia_UCG_014* may be linked to the regulation of CHOL, and jejunal *Streptococcaceae* may be involved in the regulation of TG. Additionally, microbial GWAS results indicated that the presence of gut microbiota was under host genetic regulation. Our findings provide valuable insights into the complex interaction between host genetics and microbiota in shaping the blood biochemical profile of chickens.

**Key points:**

*• Multiple candidate genes were identified for the regulation of CHOL, HDL-CH, and BG.*

*• RF39, Clostridia_UCG_014, and Streptococcaceae were implicated in CHOL and TG modulation.*

*• The composition of gut microbiota is influenced by host genetics.*

**Supplementary Information:**

The online version contains supplementary material available at 10.1007/s00253-023-12814-8.

## Introduction

Blood biochemical indicators can objectively reflect physiologic or pathological alterations, as well as the metabolic and nutritional status of an animal. Cholesterol (CHOL), triglycerides (TG), and blood glucose (BG) are important indicators affecting the growth performance of chickens through carbohydrate and lipid metabolism. In the bloodstream, cholesterol exists as lipoproteins, including high-density lipoprotein cholesterol (HDL-CH), low-density lipoprotein cholesterol (LDL-CH), and very low-density lipoprotein cholesterol (VLDL-CH). Cholesterol homeostasis is essential for cellular and systemic functions, and elevated cholesterol levels in livestock can negatively impact health and productivity, including egg production and quality, reproduction, and hatchability. Furthermore, high cholesterol levels can increase the risk of fatty liver and arteriosclerosis (Jiang et al. 1990; Luo et al. 2020). Poultry abdominal fat deposition and egg yolk formation depend on plasma triglyceride levels. Elevated triacylglycerol levels are associated with metabolic and physiological disorders such as fatty liver and insulin resistance, which can negatively affect the health and productivity of livestock (Hermier et al. 1984; Arsenault et al. 2010). Blood glucose is a crucial source of energy for animals, and changes in blood glucose levels under pathological or environmental conditions can indicate the overall health status of the organism. Blood glucose is involved in processes such as carbohydrate and lipid metabolism, which impacts the meat quality of livestock and poultry (Choe and Kim 2014).

Numerous studies have demonstrated that blood biochemical indicators are subject to genetic regulation. For instance, Cohen et al. (2005) conducted sequencing of the coding region of *PCSK9* and identified two nonsense mutations (*Y142X* and *C679X*), which were linked to a 40% reduction of LDL cholesterol in plasma (Cohen et al. 2005). Additionally, many studies investigated the polygenic basis of blood LDL-CH, HDL-CH, and TG levels using genome-wide association studies, and found the cumulative effect of multiple common genes (*ANGPTL4*, *AMAC1L2*, and *MAFB*) contributes to polygenic dyslipidemia (Kathiresan et al. 2009; Teslovich et al. 2010; Li et al. 2020). Voight et al. (2012) performed two Mendelian randomization analyses and revealed that carriers of the *LIPG 396Ser* allele exhibited higher HDL cholesterol levels (Voight et al. 2012). Moreover, research has indicated that growth traits, such as fat deposition, body weight, feed efficiency, and blood biochemistry traits, are not only influenced by host genetic regulation but also by the host’s “second genome,” i.e., the gut microbiome. The gut microbiota plays a key role in maintaining host health by altering host metabolism and impacting physical functions in both healthy and diseased states (Turnbaugh et al. 2006; Yang et al. 2015). Furthermore, the gut microbiota is capable of regulating numerous metabolic processes in the host, including energy homeostasis, glucose metabolism (Karlsson et al. 2013; Heianza et al. 2019), and lipid metabolism (Hu et al. 2022).

Genomic variations encompass various types of genetic mutations, including single-nucleotide polymorphisms (SNPs), small insertions and deletions (indels), and structural variations (SVs) (Hofmann et al. 2017). SNPs and indels have been widely used in population genetics analysis, molecular-assisted breeding, and identification of disease-related genes in animals (Wen et al. 2021). Furthermore, decades of research have shown that structural variations (SVs), such as large deletions, insertions, duplications, inversions, and translocations, can cause significant perturbations in cis-regulatory regions, resulting in quantitative changes in gene expression and phenotypes (Qin et al. 2021). As a result, SVs play a crucial role in genome diversity and are now widely used in animal breeding (Alonge et al. 2020). Currently, there is limited research on the multi-type genetic variants and multi-segmental microbiota on the regulation of blood biochemical indicators, as well as their interactions in chickens.

In this study, we measured five blood biochemical indicators and 19 growth traits of 206 yellow-feathered dwarf broilers and investigated their correlations. Using the sequencing of host whole-genome and 16S rRNA of their duodenum, jejunum, ileum, cecum, and feces microbiota, we assessed the contributions and co-regulations of host genetics and gut microbiota to blood biochemical indicators. This study will provide new perspectives and insights into understanding the growth, development, and metabolic regulation mechanisms of chickens.

## Methods

### Animals and sampling

A total of 206 male yellow-feather chickens from Guangdong Wen’s Nanfang Poultry Breeding, Co., Ltd. (Xinxing, China) were used in this study. All chickens are hatched on the same day and fed the same diet. The real-time feed intake and body weight of chickens from 56 to 76 days of age, totaling 21 days, were recorded using a feed intake measurement system, and the body weight gain (BWG), metabolic mid-bodyweight (MMBW). and residual feed intake (RFI) during this period were calculated. RFI was calculated based on the average daily feed intake, average daily gain, and metabolic mid-weight as described by Yan et al. (2019). At 78 days of age, whole blood was collected from each bird via the wing vein using a syringe, and the CHOL, LDL-CH, HDL-CH, TG, and BG contents were detected using a fully automatic biochemical analyzer (Hitachi 7020, Japan) (Yang et al. 2012). The feces were obtained from the cloaca by squeezing the abdomen. All birds were euthanized by cervical dislocation and dissected. The abdominal fat weight (AFW), breast muscle weight (BMW), leg weight (LW), and intramuscular fat (IMF) content were weighed using an electronic balance with an accuracy of 0.1 g, and abdominal fat percentage (AFWP), breast muscle percentage (BMWP), and leg weight percentage (LWP) were calculated. The sebum fat thickness (SFT) and body measurements (including body slant length (BSL), shin circumference (SC), shin length (SL), keel length (KL), and breast width (BrW)) were obtained using a tape (Supplementary Table S1). Immediately after dissection, the content (including chyme and mucosa) was collected from the duodenum, jejunum, ileum, and cecum. And all of the samples were collected in well-labeled 2 mL cryovials and rapidly placed in liquid nitrogen. Blood samples and gut content samples were transferred to a − 80 °C freezer for long-term storage.

### DNA extraction and sequencing

Host genome DNA and gut microbial DNA were extracted using the Tiangen DNA Extraction Kit (Tiangen Biotech, Beijing, China) and the QIAamp DNA stool mini kit (QIAGEN, Hilden, Germany), respectively. The host DNA library was paired-end sequenced using the Illumina Hiseq 2500 sequencing system with a length of 150 bp and depth of 10 × to ensure the stability and accuracy of the sequencing results. For microbial and fecal DNA, the region amplified was selected to be the 16S rRNA V4 region, using the specific primers 520F/802R (5’-AYTGGGYDTAAAGNG-3’ and 5’-TACNVGGGTATCTAATCC-3’), and 2 × 300 bp sequencing was performed using the Illumina Miseq sequencing system.

### Host genome sequencing data processing

Due to the failure of DNA extraction from the blood genome of one individual, a total of 205 individuals were ultimately used for genomic sequencing data processing and subsequent analysis. The chicken reference genome used in this study is version 6 (Galgal6), which was downloaded from the Ensembl website (http://ftp.ensembl.org/pub/release-106/fasta/gallus_gallus/dna/). The paired-end reads were first quality-controlled using FastQC (http://www.bioinformatics.babraham.ac.uk/projects/fastqc) software to remove primers, adapters, and low-quality reads from the library building and sequencing. Reads from the same individual and two libraries were mapped to the chicken reference genome using the default parameters of BWA v.0.7.15 (Clevenger et al. 2015) to obtain a combined SAM file. We used the SAMtools v.0.1.19 tool (Danecek et al. 2021) to sort the mapped SAM files according to the physical location of the reference genome and converted them to binary BAM files. Since PCR amplification led to a portion of reads in the final sequencing results from different clusters of the same read, and these reads were duplicated, the duplicate reads were removed using the Genome Analysis Toolkit (GATK) v.4.2.0.0 (McKenna et al. 2010), followed by file index construction using SAMtools v0.1.19.

### Microbial sequencing data processing

The paired-end reads of the microbiota were processed into amplicon sequence variants (ASVs) using the QIIME2 (ver 2020.8) (Bolyen et al. 2019) pipeline. ASV methods infer the biological sequences in the sample before the introduction of amplification and sequencing errors and distinguish sequence variants differing by as little as one nucleotide (Callahan et al. 2017). Raw fastq files were de-multiplexed based on barcode information, and then the low-quality reads were filtered with the following criteria: (1) read lengths < 150 bp; (2) contained ambiguous bases; (3) contained mononucleotide repeats > 8 bp; (4) average quality score < 20. The filtered reads were denoised using the DADA2 (The Divisive Amplicon Denoising Algorithm 2) plug-in in the QIIME2. DADA2 is a software package that models and corrects Illumina-sequenced amplicon errors. DADA2 infers sample sequences exactly, without coarse-graining into OTUs (operational taxonomic units), and resolves differences of as little as one nucleotide (Callahan et al. 2016). The sequences after DADA2 denoising are often referred to as ASVs. To further reduce data noise, this study then performed quality control on ASVs with the following criteria: (1) relative abundance > 10–6; 2) detected in more than one sample. Taxonomy analysis of ASVs based on the Silva 16S rRNA gene database v.138 (Quast et al. 2013) using classify-sklearn in QIIME2.

### SNPs and indels calling

Based on BAM files, we performed SNPs and indels calling using GATK v.4.2.0.0. To minimize false positive results, only reads with a mapping quality of more than 20 and base quality of more than 20 were used in this study for subsequent genotyping. The variants calling and genotyping were performed using the HaplotypeCaller and GenotypeGVCFs module in GATK. SelectVariants module was used to extract SNPs or indels, respectively, and only biallelic variants were extracted. Indels smaller than 50 bp were filtered out using the “–max-indel-size 50” argument. The detected SNPs and indels were strictly quality-controlled using the VariantFiltration module. The SNP quality control criteria were (1) QD > 10.0; (2) MQ > 10.0; (3) FS < 60.0; (4) MQRankSum >  − 12.5; and (5) ReadPosRankSum >  − 8.0; and the quality control standard for indels are (1) QD > 2.0; (2) FS < 200.0; (3) QUAL > 30.0; and (4) ReadPosRankSum >  − 20.0. The SNPs and indel dataset were quality controlled using PLINK v.1.9 (Purcell et al. 2007) with quality control criteria of (1) sample detection rate > 95%, (2) SNP/indel detection rate > 95%, and (3) minimum allele frequency > 1%. Then, the dataset that met the criteria was genotype-imputed using Beagle v.4.0 (Browning and Browning 2007). SNPs and indels are annotated with ANNOVAR (Wang et al. 2010) and SnpEff (Yen et al. 2017) respectively. Since there is more than one annotation reported in each variant when using SnpEff, when multiple effects are reported, we took the first-ranked annotation for statistics.

### Identification of SVs

SVs were identified through Manta v.1.6.0 (Chen et al. 2016) and DELLY v.0.8.7 (Rausch et al. 2012). The two software called SVs by performing mapped paired-end read and split read analyses and were run with default parameters to detect deletions (DEL), inversions (INV), duplications (DUP), and translocations (TRA). SV call sets from Manta and DELLY were then merged with SURVIVOR v.1.0.7 (Jeffares et al. 2017). This will merge all the vcf files specified in sample_files together using a maximum allowed distance of 1 kb, as measured pairwise between breakpoints, and only to report calls supported by 2 callers and they have to agree on the same type and the same stand of the SVs (Jeffares et al. 2017). The quality control of SVs was using the parameter –maf 0.01 –geno0.05 –mind 0.05 and the annotation used ANNOVAR and SnpEff.

### Exploring the association between blood biochemical indicators and growth traits

First, the normality of 5 blood biochemical traits and 19 growth traits were assessed using the Shapiro–Wilk test in the R program v.4.2.2 (Wan et al. 2022), and all phenotypic data was transformed using a Box-Cox normal transformation for subsequent analysis (Supplementary Table S2) (Biscay Lirio et al. 1989; Hernandez-Segura et al. 2019). In order to understand the potential association between blood biochemical indicators and growth traits, we conducted Pearson’s correlation analysis using the psych package in R to calculate the correlation coefficients between the blood biochemical traits and growth traits. *P*-value < 0.05 after BH correction is a significant threshold. We then performed bivariate REML analysis in GCTA v.1.94 (Yang et al. 2011) to estimate the genetic correlation between the blood biochemical traits and growth traits using independent SNP markers. The genetic correlation is defined as ${r}_{g}= \frac{{\sigma }_{g1g2}}{{\sigma }_{g1}{\sigma }_{g2}}$, where the subscripts “1” and “2” represent the two traits; ${\sigma }_{g1g2}$ is the genetic covariance; ${\sigma }_{g1}$ and ${\sigma }_{g2}$ are the genetic variance of traits 1 and 2, respectively. We used the likelihood-ratio test statistic to test the hypotheses that the genetic correlation coefficient is zero (no genetic correlation) and obtain accompanying *P* values (Deary et al. 2012).

### Assessment of the impact of host genetics on blood biochemical indicators

To estimate the proportion of phenotypic variance that is accounted for by genome-wide SNPs, we utilized the GREML algorithm within the GCTA to estimate the heritability of blood biochemical indicators (Yang et al. 2011; Wen et al. 2019). Subsequently, to investigate the phenotype-related host genetic variation, genome-wide association analysis was performed using univariate linear mixed models (ULMM) in GEMMA v.0.98.5 software (Zhou and Stephens 2012). GEMMA can fit a univariate linear mixed model in the following form:

$$\mathrm{y}=\mathrm{W\alpha }+\mathrm{x\beta }+\mathrm{u}+\upepsilon ;\mathrm{u}\sim \mathrm{MVN}n\left(0, {\lambda \tau }^{-1}\mathrm{K}\right),\upepsilon \sim \mathrm{MVN}n\left(0,{\tau }^{-1}\mathrm{I}n\right)$$

where **y** is an *n*-vector of quantitative traits (or binary disease labels) for *n* individuals; **W** = (**w**_1_, · · ·, **w**_c_) is an *n* × *c* matrix of covariates (fixed effects) including a column of 1 s; ***α*** is a *c*-vector of the corresponding coefficients including the intercept; **x** is an *n*-vector of marker genotypes; β is the effect size of the marker and is an estimate of the marker additive effect; **u** is an *n*-vector of random effects; ${\varvec{\upepsilon}}$ is an *n*-vector of errors; τ^−1^ is the variance of the residual errors; λ is the ratio between the two variance components; **K** is a known *n* × *n* relatedness matrix and **In** is an *n* × *n* identity matrix. MVN*n* denotes the *n*-dimensional multivariate normal distribution. The significance *p-*value level between SNPs, indels, or SVs and phenotypes was calculated from a derived score test.

To control the effect of population structure on GWAS analysis, we first constructed relatedness matrices based on different variant types. Specifically, if variants with lower minor allele frequency tend to have larger effects, then the standardized genotype matrix is preferred. Therefore, the relatedness matrices were first estimated from the standardized genotype information of SNPs, indels, and SVs respectively. The relatedness matrices were calculated as follows:

$$Gs=\frac{1}{p}\sum_{i=1}^{p}\frac{1}{{v}_{xi}}({\mathbf{x}}_{i}-{1}_{n}{\overline{x} }_{i}){({\mathbf{x}}_{i}-{1}_{n}{\overline{x} }_{i})}^{T}$$

We denote **X** as the *n* × *p* matrix of genotypes, x_*i*_ as its *i*th column representing genotypes of the *i*th variant, ${\overline{x} }_{i}$ as the sample mean and ${v}_{xi}$ as the sample variance of *i*th variant, and **1**_*n*_ as a *n* × 1 vector of 1’s. Then, the correlation was calculated for the relatedness matrices composed of different variant types. Since the relatedness matrices constructed based on SNPs and indels have high similarity, and the relatedness matrix constructed based on SVs has low similarity with the other two types with the number of detected SVs is small, it is difficult for SVs to capture complete genetic variation information. Therefore, subsequent GWAS with SNPs and indels were conducted with their own relatedness matrix for correction, while GWAS of SVs were corrected using the relatedness matrix constructed based on SNPs. The effect of population stratification was corrected by adjusting the first five principal components (PCs) as derived from the whole-genome SNPs and indels (Price et al. 2010). To avoid potential false positives in multiple comparisons, the whole-genome significance threshold was adjusted via the Bonferroni adjustment (Sedgwick 2014). For SNPs, we first calculated their valid independent count using simpleM (Wen et al. 2021), which was determined to be 1,535,191. Therefore, we set the significance thresholds as − log_10_(0.05/1,535,191) = 7.49. For indels and SVs, we set the thresholds as − log_10_(0.05/1,332,016) = 7.43 and − log_10_(0.05/13,303) = 5.42, respectively. In addition, the Manhattan plots and quantile–quantile (Q-Q) plots of the MLM for individual traits were implemented in R (Gacesa et al. 2022).

### Investigating the correlation between gut microbiota and blood biochemical indicators

To determine the effect of gut microbiota from different intestinal segments on phenotype, we estimated the microbiability of five blood biochemical indicators based on microbiota from different segments of the intestine. The proportion of the total variance explained by the gut microbiota is called microbiability (Wen et al. 2021) and is defined as *m*^2^ = *σ*^2^_*m*_/*σ*^2^_*p*_, where *σ*^2^_*m*_ is the microbial variance. We first constructed a microbial relationship matrix (MRM) based on the *Z*-score standardized matrix of ASV relative abundance, and then used the REML algorithm in GCTA to calculate the microbiability (Wen et al. 2019) based on the MRM, The formula for calculating the MRM matrix is as follows:

$${\mathbf{M}}_{s}=\frac{{\mathbf{X}}_{s}{\mathbf{X}}_{s}^{T}}{{\mathrm{N}}_{s}}$$

${\mathbf{M}}_{s}$ is the MRM of the gut segment s, ${\mathbf{X}}_{s}$ is the normalized relative abundance matrix of the taxa, ${\mathbf{X}}_{s}^{T}\mathrm{ is the transpose of}{\mathbf{X}}_{s}$, and Ns is the number of taxa used to estimate the MRM in gut segments (Wen et al. 2019, 2021).

To investigate the specific families associated with blood biochemical indicators, we performed a correlation analysis between families with detection rates > 10% and blood biochemical indicators. Families with detection rates > 50% were considered as continuous phenotypic traits, and the relative abundances were subjected to inverse normal transformation, where all zero counts were turned into missing values (represented by NAs) (Hughes 2020; Grieneisen et al. 2021). Spearman’s correlation analysis was performed between the transformed relative abundances and blood biochemical indicators. A *P*-value < 0.05 after false discovery rate (FDR) correction was considered to be significantly correlated. Families with detection rates between 10 and 50% were considered as presence/absence (P/A) phenotypes, with presence (relative abundance > 0) coded as 1 and absence (relative abundance = 0) coded as 0. Then, polyserial correlation analysis was performed for these families. A *P*-value < 0.05 indicates a significant association between the presence/absence of a family and a blood biochemical indicator. Subsequently, a two-tailed analysis was performed on the families with a correlation coefficient greater than 0.2 among the families with a detection rate > 50%. We sequentially selected the top and bottom 20% individuals based on blood biochemical indicators or microbial abundance, and performed Wilcoxon’s rank-sum test on microbial abundance or blood biochemical indicators (Mahajan et al. 2018). If there were significant differences (*P* < 0.05) in blood biochemical indicators between high and low microbial abundance groups, as well as significant differences (*P* < 0.05) in microbial abundance between high and low blood biochemical indicator groups, then an association was considered to exist between the blood biochemical indicator and the microbiota.

### Exploring the role of host genetics in regulating gut microbiota

To investigate the impact of host genetics on the gut microbiota, we performed a microbial genome-wide association study (mGWAS) analysis using microbial relative abundance as a phenotype with the GEMMA software. Our mGWAS analysis was based on SNPs, indels, and SV information. We classified the families into continuous and P/A types based on their detection rate. Families with a detection rate greater than 50% were categorized as continuous types, and their relative abundances were transformed using inverse normal transformation before analysis. Families with a detection rate between 10 and 50% were classified as P/A types, with 1 indicating presence (relative abundance > 0) and 0 indicating absence (relative abundance = 0). The *P* values were adjusted using the Bonferroni method. A significant *P*-value for continuous traits indicated that the relative abundance of the family was genetically regulated, whereas for P/A traits, a significant *P*-value indicated that the presence or absence of the family was genetically regulated. Subsequently, in order to further investigate the interplay among host genetics, gut microbiota, and blood biochemical traits, we selected bacterial taxa with significant loci in both SNPs, indels, and SV mGWAS analyses. We then searched for shared loci or genes between mGWAS and GWAS. Additionally, we focused on the significant loci or genes from SNPs, indels, and SV mGWAS within these bacterial taxa. Through gene functional annotation and in combination with the correlation analysis between these bacterial taxa and blood biochemical indicators, we identified genes and bacterial taxa that co-regulate blood biochemical indicators.

## Results

### Association between blood biochemical indicators and chicken growth traits

**Fig. 1:**
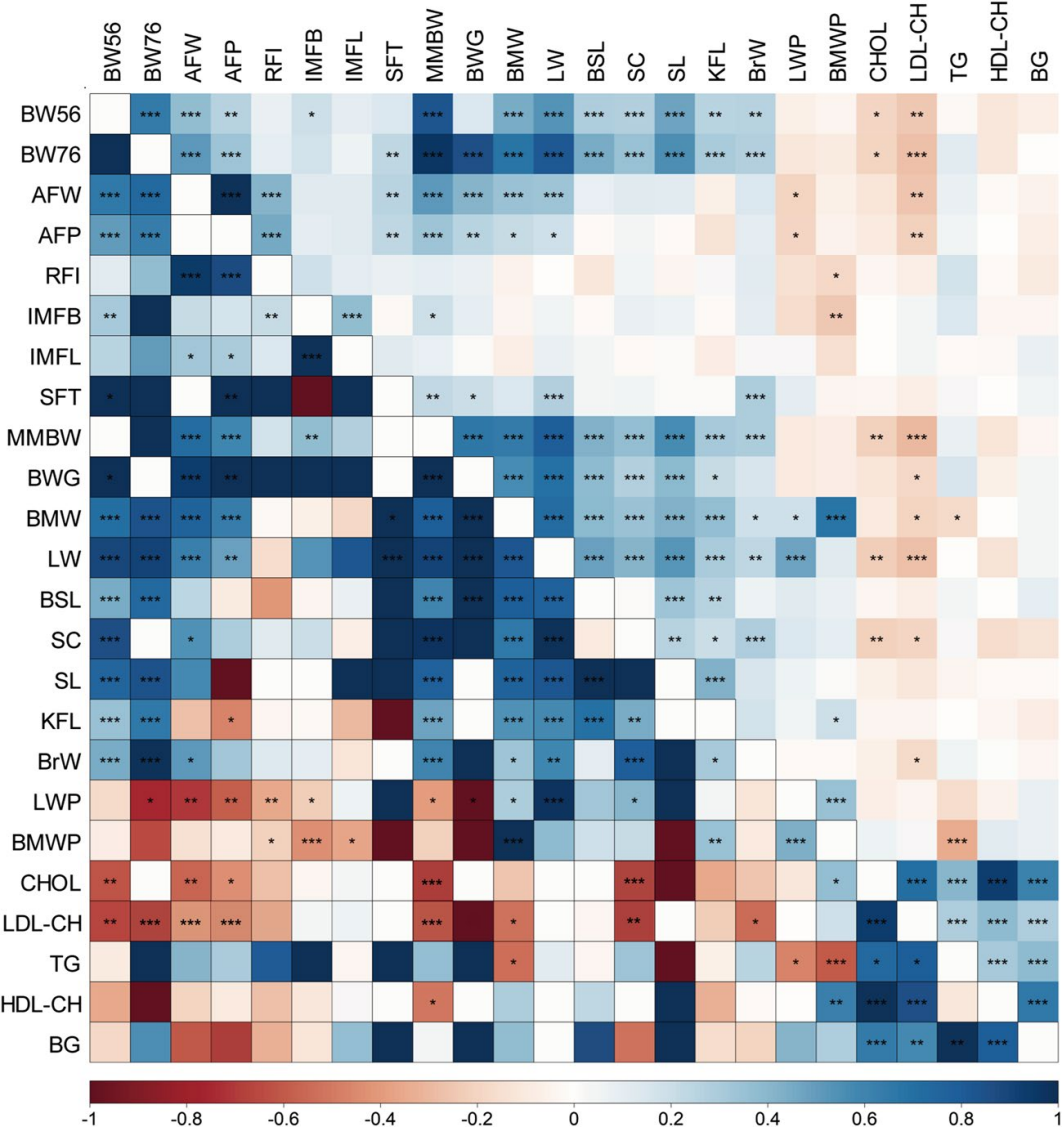
The correlation between 24 traits. The upper triangle is Pearson’s phenotypic correlation analysis, the lower triangle is genetic correlation analysis. The symbols *, **, and *** represent *P* values < 0.05, 0.01, and 0.001, respectively. Red indicates negative correlations; blue indicates positive correlations

In this experiment, 24 traits were measured in chickens, including 5 blood biochemical traits, i.e., total cholesterol (CHOL), low-density lipoprotein cholesterol (LDL-CH), triglycerides (TG), high-density lipoprotein cholesterol (HDL-CH), and blood glucose (BG) and 19 growth traits (Supplementary Table S1). The descriptive statistics were performed for the five blood biochemical traits, and it was observed that their coefficients of variation were between 17.27 and 31.87%, indicating a large variability in this population (Table 1). Pearson’s correlation analysis indicated that CHOL, LDL-CH, and TG were significantly negatively correlated with growth traits such as body weight (BW), abdominal fat weight (AFW), abdominal fat percentage (AFP), metabolic mid-bodyweight (MMBW), body weight gain (BWG), breast muscle weight (BMW), leg weight (LW), shin circumference (SC), and breast muscle weight percentage (BMWP) (Table 2). The correlation among blood biochemical traits was all positive, with the highest correlation coefficient between CHOL and HDL-CH (*r* = 0.92, *P*_adj_ < 0.001), followed by that between CHOL and LDL-CH (*r* = 0.7, *P*_adj_ < 0.001), suggesting a certain degree of synergistic effect of blood biochemical indicators (Fig. 1, Supplementary Table S3). To examine the extent to which the correlation in these traits can be attributed to genetic factors, we estimated the genetic correlations between blood biochemical indicators and growth traits. The results showed that the genetic correlation coefficients among blood biochemical indicators and growth-related traits were generally consistent with the phenotypic correlations. Specifically, CHOL and LDL-CH were significantly and negatively correlated with BW, AFW, AFP, MMBW, BMW, and SC (rG =  − 0.40 ~  − 0.67, *P* < 0.05) (Supplementary Table S3), suggesting the indicative role of CHOL and LDL-CH on growth traits in chicken.

**Table 1:** Descriptive statistics for blood biochemical indicators

Traits	Observation number	Mean ± SD	CV %	Max	Min
CHOL (mmol/L)	205	3.44 ± 0.73	21.22	8.90	1.56
LDL-CH (mmol/L)	205	0.91 ± 0.29	31.87	2.27	0.31
TG (mmol/L)	205	0.33 ± 0.10	29.00	0.79	0.15
HDL-CH (mmol/L)	205	2.55 ± 0.57	22.36	7.10	1.06
BG (mmol/L)	205	11.87 ± 2.05	17.27	24.69	3.00

Note: *CHOL* cholesterol, *LDL-CH* low-density lipoprotein cholesterol, *TG* triglycerides, *HDL-CH* high-density lipoprotein cholesterol, *BG* blood glucose, *SD* standard deviation, *CV* variation coefficient

**Table 2:** Pearson’s correlation of blood biochemical indicators with growth traits

	CHOL	LDL-CH	TG	HDL-CH	BG
BW56	− 0.20*	− 0.26**	− 0.03	− 0.12	− 0.08
BW76	− 0.21*	− 0.28**	0.11	− 0.12	− 0.01
AFW	− 0.13	− 0.26**	0.07	− 0.02	− 0.06
AFP	− 0.11	− 0.22**	0.05	− 0.01	− 0.08
MMBW	− 0.22**	− 0.30**	0.07	− 0.13	− 0.04
BWG	− 0.13	− 0.19**	0.16	− 0.07	0.04
BMW	− 0.11	− 0.21**	− 0.18	− 0.01	0.03
LW	− 0.23**	− 0.28**	− 0.01	− 0.14	0.03
SC	− 0.22**	− 0.20*	0.02	− 0.17	− 0.14
BMWP	0.06	0.02	− 0.34**	0.11	0.07

Note: * indicates a significant level of correlation (*P*_adj_ < 0.05), ** indicates a highly significant level of correlation (*P*_adj_ < 0.01). *BW56* body weight at 56 days of age, *BW76* body weight at 76 days of age, *AFW* abdominal fat weight, *AFP* abdominal fat percentage, *MMBW* metabolic mid-bodyweight, *BWG* body weight gain, *BMW* breast muscle weight, *LW* leg weight, *SC* shin circumference, *BMWP* breast muscle weight percentage

### Genetic regulation of blood biochemical indicators in chicken

**Fig. 2:**
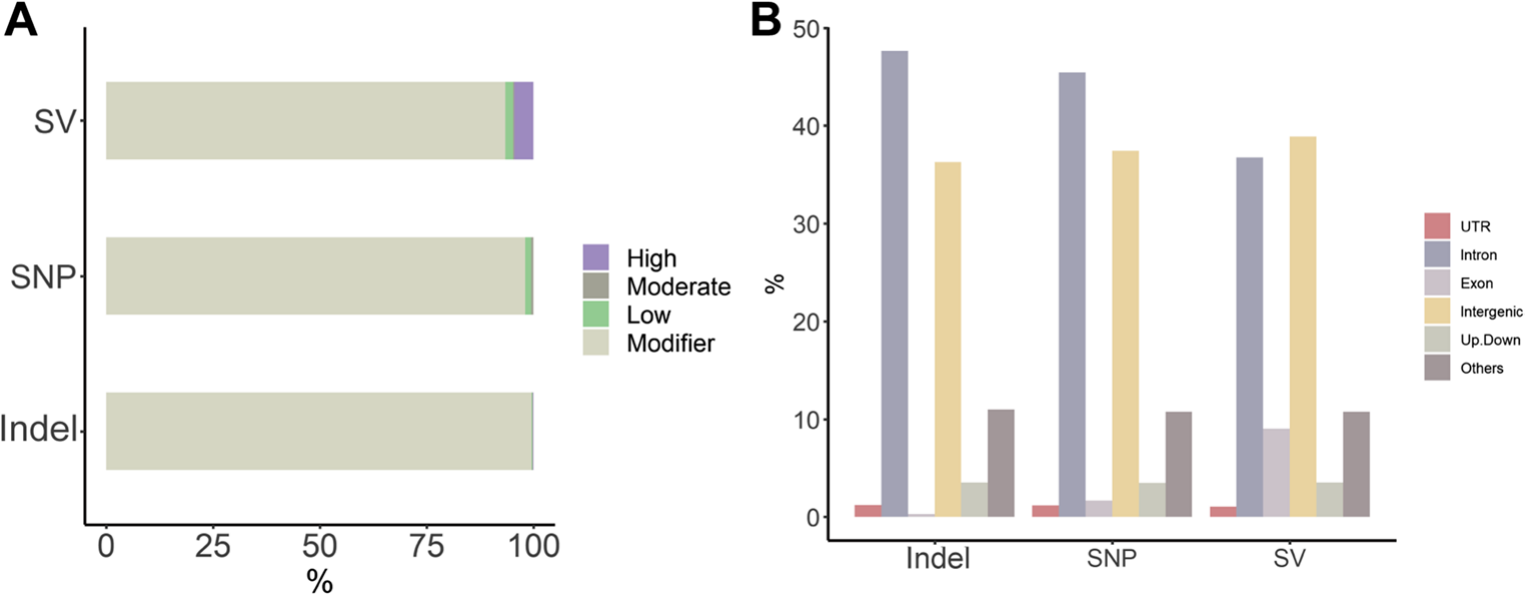
The annotation results for different variants. **A** The number of effects by impact for all types of variants. **B** The number of effects by type and region

After a series of quality control, we finally obtained 3,803,923 SNPs and 1,332,016 indels (2 ~ 50 bp). In addition, a total of 13,303 SVs (> 50 bp) were identified, including 11,583 deletions, 1150 duplications, 552 translocations, 17 conversions, and 6 insertions. First, the effects of these genetic variants were evaluated using SnpEff. Only a small fraction of variants (6.67% of SVs) had significant effects ranging from low to high, while 93.33% of SVs were classified as modifiers (no effects). For SNPs and indels, over 98% were identified as modifiers, indicating that the effects of the majority of variants were negligible (Fig. 2A, Supplementary Table S4). Subsequently, using ANNOVAR to classify the effective variants by type and region, we found that intronic and intergenic variations were much higher than other types, totally accounting for more than 75%. While variations in exons accounted for less than 10%, with 0.30% in indels, 1.67% in SNPs, and 9.01% in SVs (Fig. 2B, Supplementary Table S5). This may be due to the fact that the variations in large segments cover a more extensive region and are more prone to cause exon changes. The integration of variants is shown in Table 3.

**Table 3:** Descriptive statistics of all variants in functional regions

	Type	Count		Percent
Gene region	3’UTR	48,511	3,055,584	59.34%
	5’UTR	13,118		
	3’UTR; 5’UTR	313		
	Intronic	2,368,691		
	Exonic	68,784		
	ncRNA	555,760		
	Splicing	407		
Non-gene region	Intergenic	1,913,254	2,093,589	40.66%
	Upstream	85,413		
	Downstream	87,310		
	Upstream; downstream	7612		

**Fig. 3:**
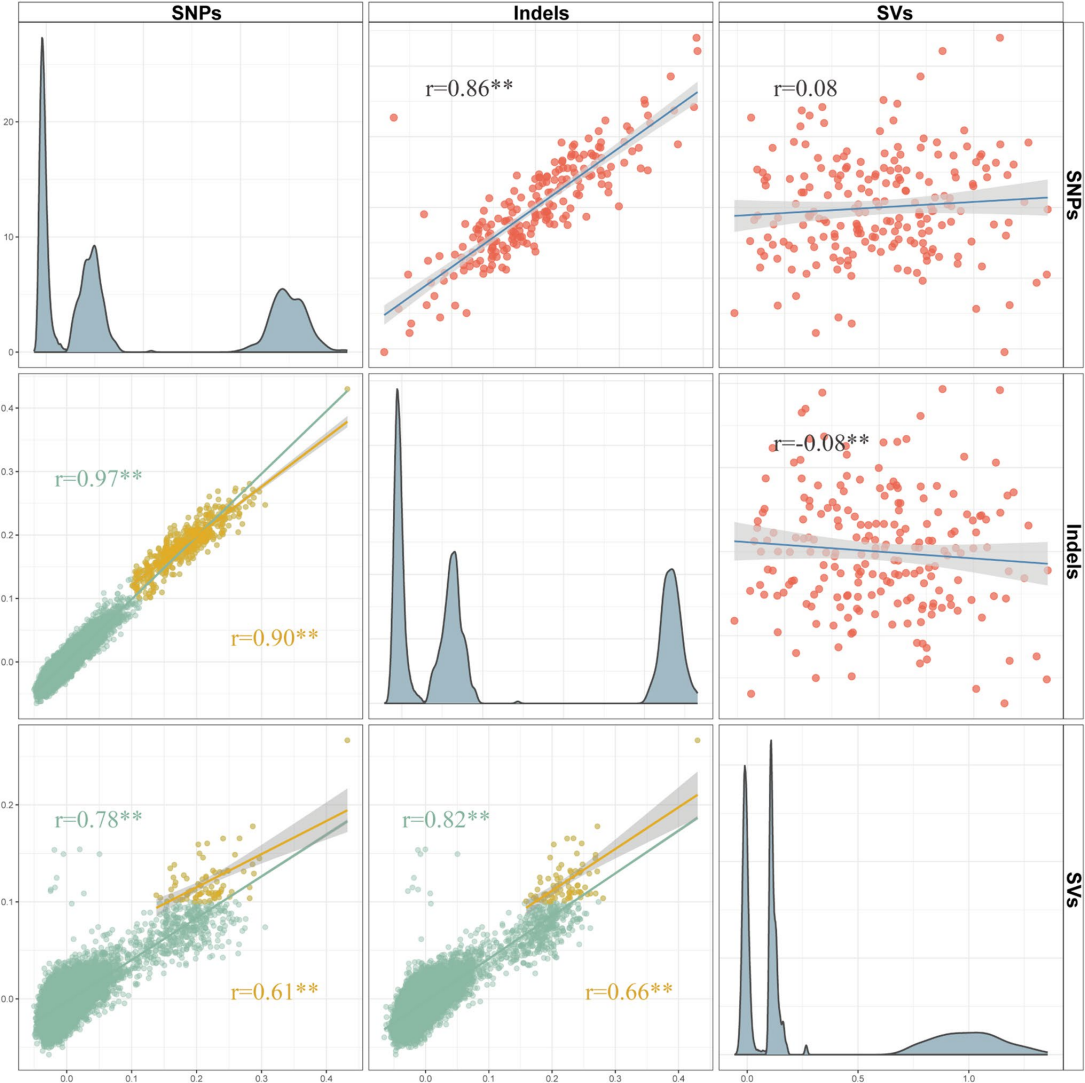
Correlations of relatedness matrices based on SNPs, indels, and SVs. The upper triangle represents the correlation of relatedness coefficients within the same individual. The lower triangle represents the correlation of relatedness coefficients between different individuals, with green indicating the correlation of relatedness coefficients between all different individuals, and yellow indicating the correlation between individuals with relatedness coefficients greater than 0.1. The diagonal shows the density plot of relatedness coefficients. *r* denotes the correlation coefficient of relatedness matrices, with ** indicating a *P*-value < 0.01

To explore the effects of host genetics on phenotypes, we first estimated the heritability of these five blood biochemical traits based on whole-genome SNP variants. The results showed CHOL, LDL-CH, HDL-CH, and BG were heritable, with heritability of 0.23, 0.19, 0.24, and 0.15. However, the heritability of TG is negligible (0.03), indicating that plasma TG levels were highly susceptible to the internal metabolic state of the body (Supplementary Table S6). Then, we performed GWAS analysis for 5 blood indicators. To correct the effect of population structure, we firstly constructed relatedness matrices based on SNPs, indels, and SVs respectively. The results showed that the relatedness matrices constructed by SNPs and indels have a high similarity (within-individual: *r* = 0.86, between-individuals: *r* ≥ 0.9). However, their correlation with the matrix established by SVs was relatively weak (within-individual: *r* < 0.1, between-individuals: *r* = 0.61 ~ 0.82, Fig. 3). This can be attributed to inadequate sequencing depth and limited detection of SV variations, making SVs unsuitable for constructing the relatedness matrix. As such, subsequent GWAS for SNPs and indels were performed with their own relatedness matrix for correction, while GWAS for SVs were corrected using the relatedness matrix constructed based on SNPs.

**Fig. 4:**
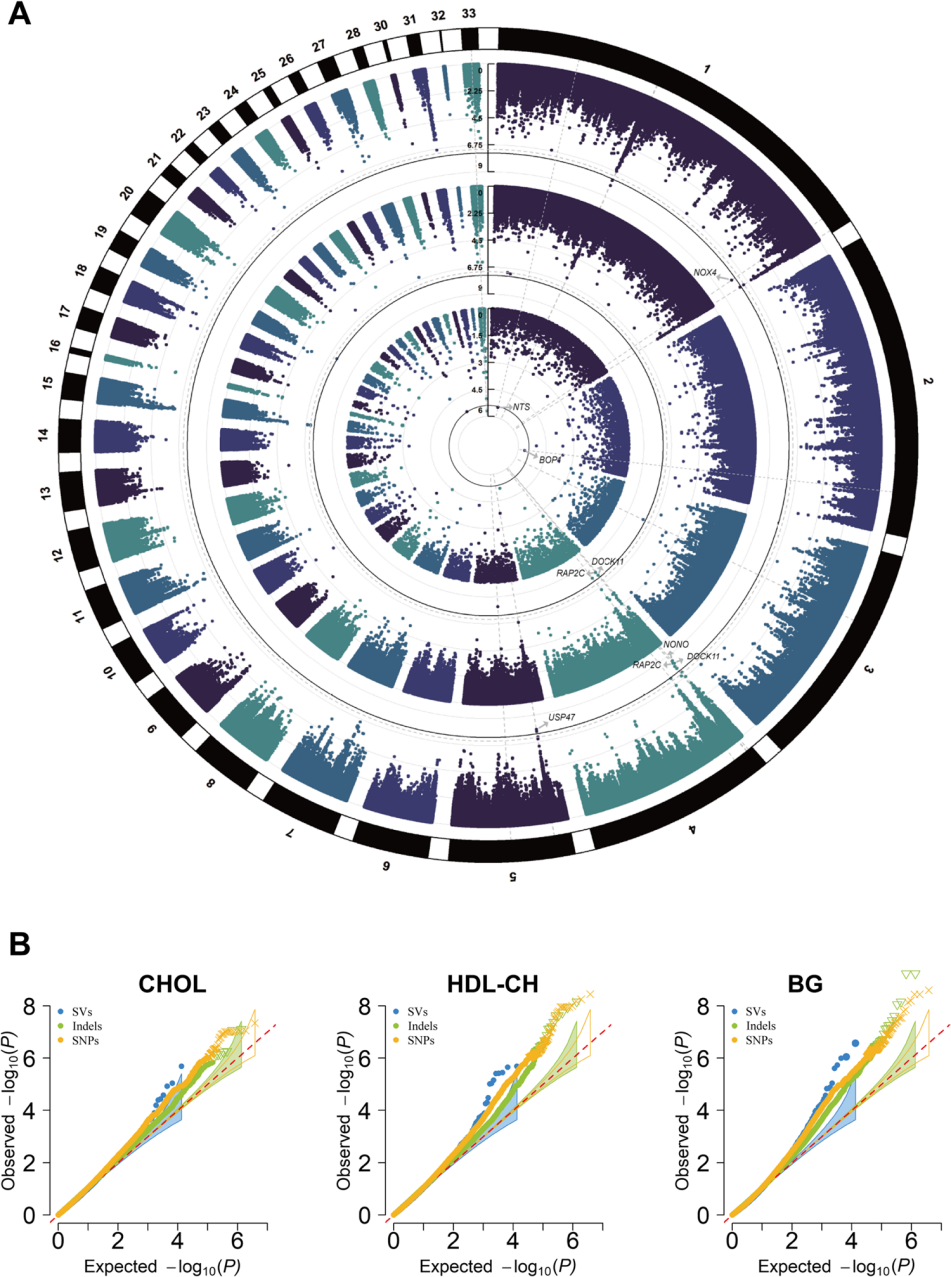
GWAS analysis of HDL-CH. **A** The Circular-Manhattan plot of HDL-CH. From the outer circle to the inner circle are the SNPs, indels, and SVs-GWAS. The horizontal black solid and grey dashed lines indicate genome-wide significance and suggestive significance thresholds (for SNPs, significant and suggestive significant thresholds were 3.26 and 6.51; for indel were 3.76 and 7.51; for SVs, significant threshold was 5.42). **B** The QQ plot of CHOL, HDL-CH, and BG

The GWAS analysis was performed on 5 blood biochemical indicators with the genotyping data of SNPs, indels, and SVs, and the results revealed significant loci for their association with CHOL, HDL-CH, and BG. The GWAS analysis for HDL-CH identified 16 SNPs, 5 indels, and 3 SVs that were statistically significant (Fig. 4A). These 16 SNPs and 5 indels were annotated to 12 and 5 genes respectively, with two shared genes, *DOCK11* and *RAP2C*, further suggesting their regulatory roles for HDL-CH. Additionally, two deletions of 94 bp and 578 bp were identified by the SVs-GWAS, located upstream of the *NTS* gene on chromosome 1 and within the intron of the *BOP1* gene on chromosome 2, respectively, both of which were associated with cholesterol regulation (Supplementary Tables S7–S10). For CHOL, a suggestive significant locus rs734932526 on chromosome 4 was detected in SNPs-GWAS and a deletion of 263 bp on chromosome 33 was detected in SVs-GWAS. Gene annotation results indicated that both loci reside on novel genes (*ENSGALG00000053344* and *ENSGALG0000004756*5) (Supplementary Fig. S1A, Supplementary Tables S7 and S9); for BG, a total of 21 significant loci (10 SNPs, 6 indels, and 5 SVs) were detected. Among the genes annotated by these loci, *GDPD5*, *DHDH*, and *KCNIP1* genes were considered as the candidate genes for their involvement in lipid and glucose metabolism (Supplementary Fig. S1B, Supplementary Tables S7, S9, and S10). The above results indicate that blood biochemical indicator levels are jointly regulated by SNPs, indels, and SVs, among which CHOL is regulated by *DOCK11*, *RAP2C*, *NTS*, and *BOP1*, and BG is associated with *GDPD5*, *DHDH*, and *KCNIP1* genes.

### Regulation of blood biochemical indicators by gut microbiota

The final number of samples used for microbial 16S rRNA sequencing was 1026 due to insufficient fecal sample collection from four individuals (cecum, duodenum, ileum, jejunum: 206, feces: 202). A total of 75,029 features were identified by ASV analysis of sequencing data, and a total of 840 species, 691genera, 365 families, 225 orders, 102 classes, and 41 phyla were obtained (Supplementary Table S11).

**Fig. 5:**
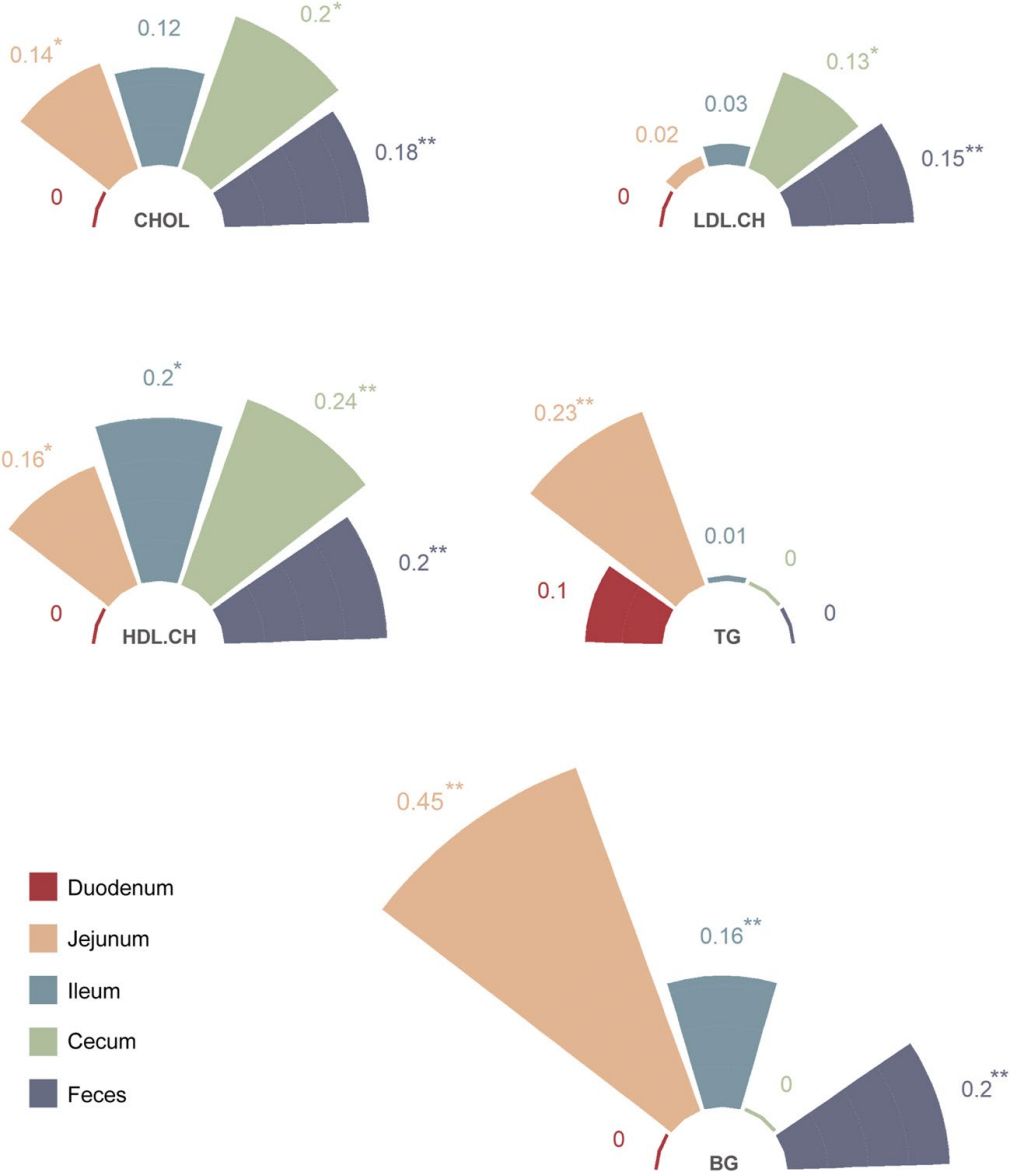
Microbiability of five blood biochemical traits. * stands for suggestively significant (*P* < 0.1); ** stands for significant (*P* < 0.05)

To assess the effects of gut microbiota from different intestinal segments on the blood biochemical indicators, the microbiabilities were calculated (Fig. 5, Supplementary Table S12). The microbial regulation patterns of CHOL and HDL-CH were found to be similar, with the duodenal microbiota having almost no regulatory effect (*m*^*2*^ = 0), while microbiota in other intestinal segments exhibited varying degrees of regulatory effects (*m*^*2*^ = 0.12 ~ 0.24), with the cecum playing the most significant regulatory role (*m*^*2*^ = 0.2, 0.24). The regulation of BG and TG is mainly controlled by the jejunal microbiota, with *m*^*2*^ reaching 0.45 and 0.23, respectively. The regulatory effects of each intestinal segment on LDL-CH were weak, with only the cecum and feces having slightly higher effects (*m*^*2*^ = 0.13, 0.15). In summary, CHOL, LDL-CH, and HDL-CH levels are primarily regulated by the cecum, while TG and BG levels are mainly regulated by the jejunal microbiota.

**Fig. 6:**
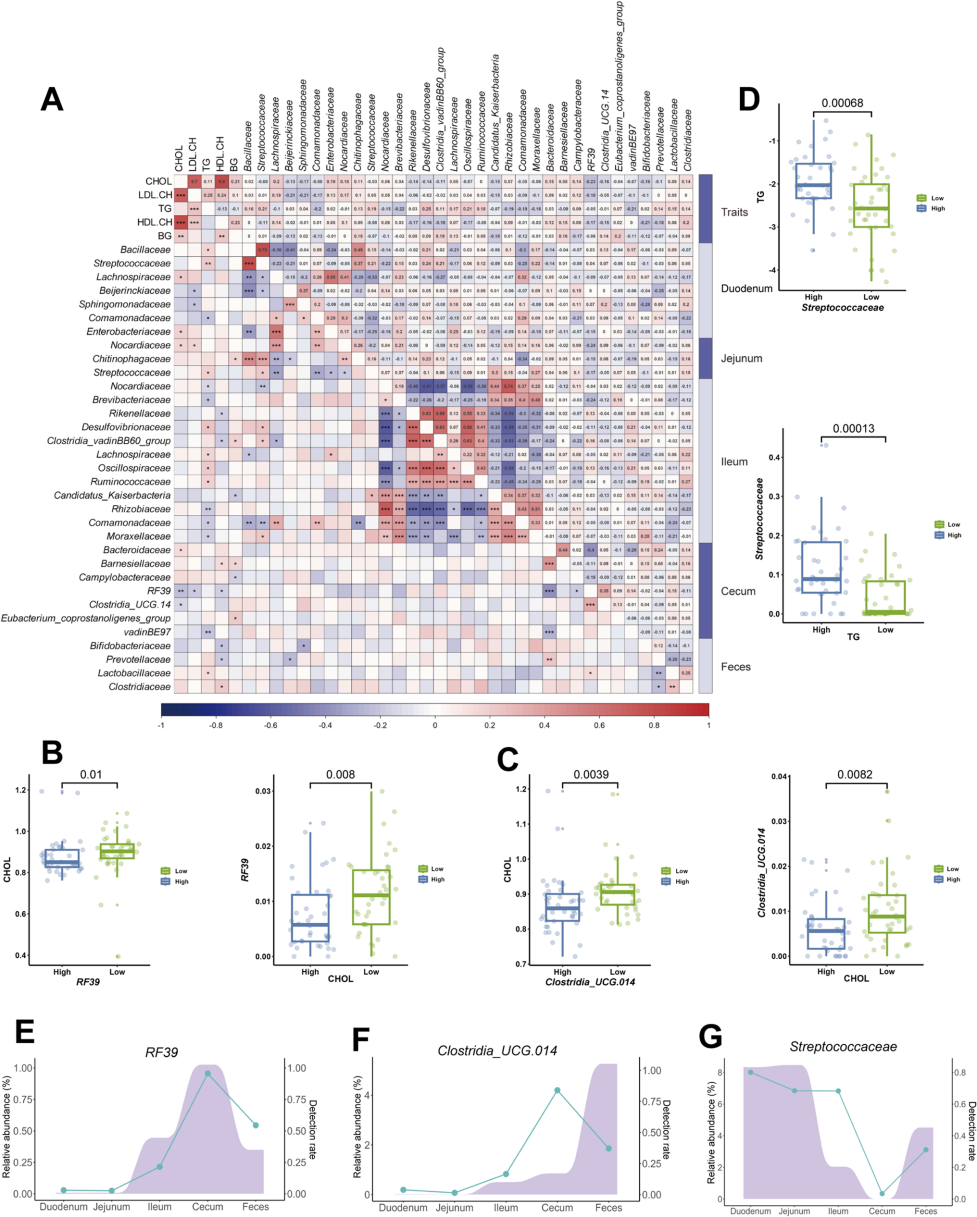
The relationship between families with a detection rate greater than 50% and blood biochemical indicators. **A** Spearman’s correlation analysis of blood biochemical indicators with the detection rate of > 50% of families. Families with |r|> 0.2 are shown. The upper triangle is the correlation coefficient, and the lower triangle is the *P*-value (* means *P* < 0.1, ** means *P* < 0.05, *** means *P* < 0.01). **B** Differences in the CHOL (*RF39*) between the two groups with the highest and lowest *RF39* abundance (CHOL). **C** Differences in the CHOL (*Clostridia_UCG.014*) between the two groups with the highest and lowest *Clostridia_UCG.014* abundance (CHOL). **D** Differences in the TG (*Streptococcaceae*) between the two groups with the highest and lowest *Streptococcaceae* (TG). **E–G** Relative abundance and detection rate of *RF39*, *Clostridia_UCG.014*, and *Streptococcaceae* in different intestinal segments. The purple represents the relative abundance, and the blue line represents the detection rate

To further identify the specific microbes that associated with blood biochemical indicators, the families with detection rates > 50% were used for Spearman’s correlation analysis. Only the microbes with at least one blood biochemical indicator having a |r|> 0.2 and *P* < 0.05 were retained, resulting in a total of 33 microbes (7 in duodenum, 3 in jejunum, 12 in ileum, 7 in cecum, and 4 in feces, Supplementary Table S13). As a result, weak to moderate correlations (*r* =  − 0.23 ~ 0.25) between gut microbes and five blood biochemical indicators were observed (Fig. 6A). To validate their correlation, we performed a two-tailed analysis for gut microbes with these 33 microbes. We compared the differences in family abundances between individuals with blood biochemical indicators in the top and bottom 20% (*N* = 40), as well as the differences in blood biochemical indicator levels between individuals with family abundances in the top and bottom 20% (*N* = 40) in sequence. Finally, the associations of two families *RF39* and *Clostridia_UCG.014* in the cecum with CHOL, and *Streptococcaceae* in the jejunum with TG were confirmed. In specific, the individuals with the lowest 20% of relative abundances of *RF39* and *Clostridia_UCG.014* in the cecum had significantly higher CHOL levels (*P* = 0.01, 0.0039) than the top 20% individuals. Meanwhile, those in the lowest 20% of CHOL levels had significantly higher relative abundances of *RF39* and *Clostridia_UCG.014* (*P* = 0.008, 0.0082) (Fig. 6B, C). In addition, the TG level was significantly lower in the 20% of chickens with the lowest relative abundance of jejunal *Streptococcaceae* than in the 20% with higher relative abundance (*P* = 0.00068). Correspondingly, chickens with low relative abundance of jejunal *Streptococcaceae* had a lower TG level (*P* = 0.00013) (Fig. 6D). Therefore, these results suggested that *RF39* and *Clostridia_UCG.014* in the cecum were associated with CHOL, and jejunal *Streptococcaceae* was associated with TG. We further analyzed the detection rate and relative abundance of *RF39*, *Clostridia_UCG.014*, and *Streptococcaceae* in different gut segments. The results showed that *RF39* and *Clostridia_UCG.014* were mainly detected in the cecum, with detection rates of 0.96 and 0.84, and *Streptococcaceae* was mainly colonized in the duodenum with a detection rate of 0.8. The relative abundances of *RF39* and *Clostridia_UCG.014* in all intestinal segments were with an average abundance of 0.37% and 1.33%, respectively, while the relative abundance of *Streptococcaceae* can reach up to 8.47% in the jejunum (Fig. 6E–G).

**Fig. 7:**
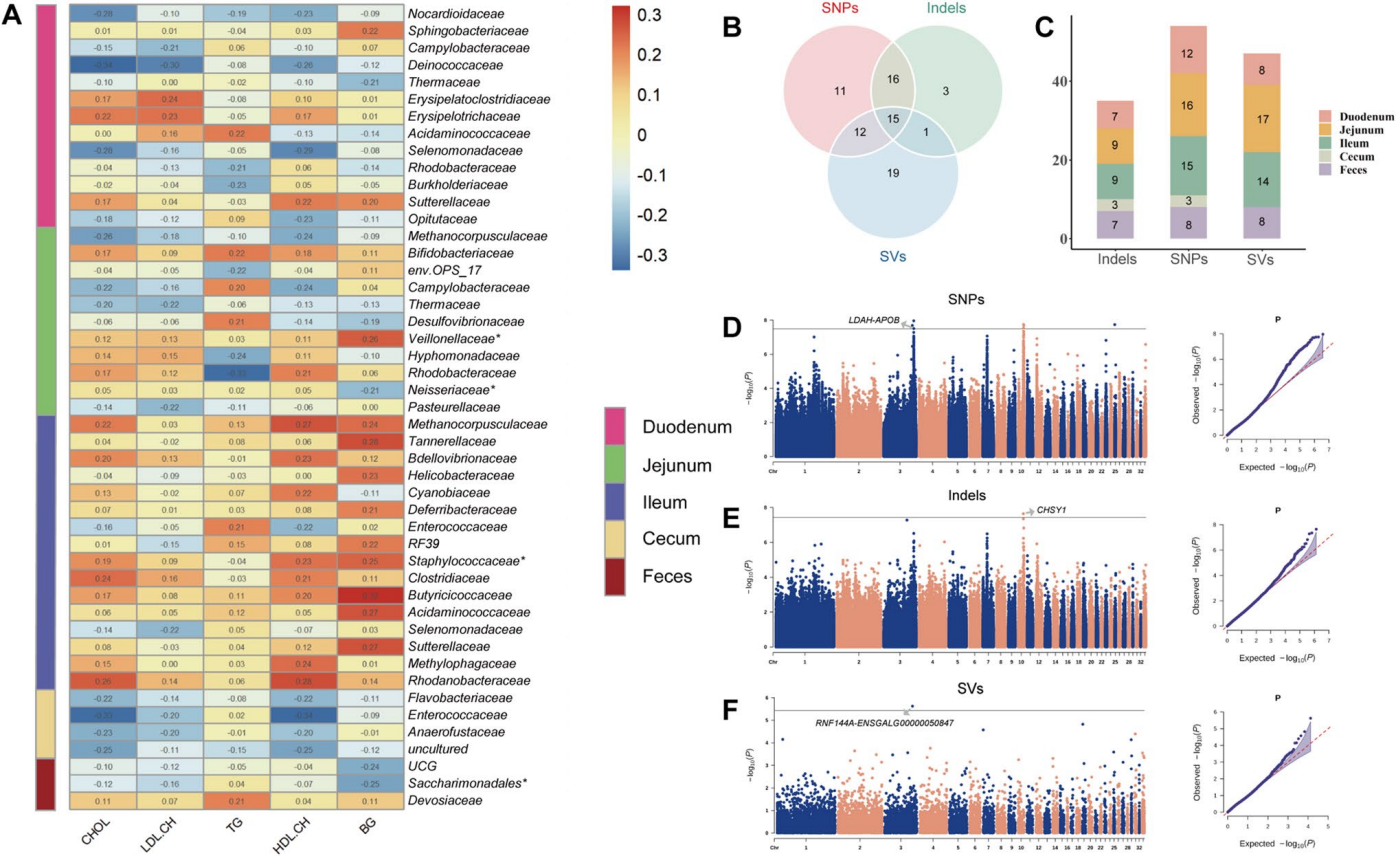
Polyserial correlation and mGWAS of P/A families. **A** Polyserial correlation between blood biochemical indicators and P/A families. The number in the color represents the correlation coefficient and families with |r|> 0.2 are shown. * indicates that significant loci were detected in these families in both SNPs, indels, and SV mGWAS. **B** The number of families with significant loci detected by SNPs, indels, and SV mGWAS. **C** The distribution of P/A traits with significant loci in different gut segments and feces. **D–F** are the Manhattan and QQ plots of the SNPs, indels, and SV mGWAS results of ileal *Staphylococcaceae.* The horizontal black lines indicate significance thresholds

As to the families with detection rates within 10 ~ 50%, polyserial correlation analysis was performed and retained only the microbes that had a significant correlation coefficient with at least one blood biochemical indicator greater than 0.2 (47 microbes, Supplementary Table S14). The correlation between P/A microbiota and phenotypes was also found to be in the range of low to moderate correlation (− 0.34 to 0.32). The polyserial correlation analysis indicated a generally positive correlation between the presence of families in the ileum with blood biochemical indicators, with the largest correlation coefficient of 0.32 observed between *Butyricicoccaceae* and BG. Meanwhile, negative correlations were observed in most of the microbes in the duodenum, jejunum, and cecum, with *Enterococcaceae* in the cecum exhibiting a correlation coefficient of − 0.34 with HDL-CH (Fig. 7A).

### The co-regulation of gut microbiota and host genetics

To explore the potential correlation between host genetics and gut microbiota, we performed SNPs, indels, and SV mGWAS analysis by treating the relative abundance of families in different gut segments as phenotypes. We found that none of the three mGWAS detected significant loci for continuous traits. However, for P/A traits, SNPs, indels, and SV mGWAS detected significant loci in 54, 35, and 47 families, respectively. Among them, 15 families were detected in all three types of mGWAS (Fig. 7B, C). This suggests that the presence of different microbes in different gut segments is regulated by multiple types of genetic variations. Subsequently, we searched for families within these 15 bacterial taxa where the mGWAS results overlapped with the GWAS results for blood biochemical indicators. Unfortunately, we were unable to identify families with overlapping loci. However, it is noteworthy that among these 15 families, jejunal *Veillonellaceae* and *Neisseriaceae* showed significant positive and negative correlations with BG (*r* = 0.26 and − 0.21), respectively, while *Staphylococcaceae* in the ileum exhibited significant positive correlations with both CHOL, HDL-CH, and BG (*r* = 0.19, 0.23, and 0.25, Fig. 7A). We further analyzed the genetic loci of these three families and annotated the candidate genes (Supplementary Tables S15–S17). It is worth noting that for ileal *Staphylococcaceae*, SNP-mGWAS detected a variant located in the intergenic region between the *LDAH* and *APOB* genes at 3_102093230 (Fig. 7D–F), both of which have been extensively reported to be directly involved in cholesterol regulation. Additionally, for jejunal *Veillonellaceae*, SNPs and SV mGWAS identified significant loci that annotate to *TOX2*, and *EDEM2*, respectively, which are also related to blood glucose regulation (Supplementary Fig. S2A–C). For jejunal *Neisseriaceae*, both SNPs and SV mGWAS detected common genes in chromosome 1, but these genes are novel genes with no functional description yet (Supplementary Fig. S2D–F). Therefore, it can be inferred that there exists an interaction between *Staphylococcaceae* and the candidate genes *LDAH* and *APOB* in regulating cholesterol levels, and there may be a certain relationship between *Veillonellaceae* and *TOX2* and *EDEM2* in regulating blood glucose levels.

## Discussion

To date, numerous studies have detected the genetic variants and genes that associated with blood biochemical indicators through various methods. These genes mainly affect the rate of metabolic pathways by influencing enzyme activity or expression, such as the involvement of hydroxy-3-methylglutaryl coenzyme A reductase (HMGCR), acetoacetyl-CoA thiolase (ACAT), and low-density lipoprotein receptor (LDLR) in cholesterol metabolism (Montoudis et al. 2008; Caselli et al. 2011; Ference et al. 2017), and the participation of insulin action and glucose uptake pathway in blood glucose metabolism (Duncan et al. 2019; Chen et al. 2022). In recent years, the impact of gut microbiota on host metabolism and phenotypic variations has been widely studied. Short-chain fatty acids (SCFAs) such as acetate, propionate, and butyrate can affect glucose and lipid metabolism when produced by gut microbiota. In addition, gut microbiota can influence inflammation and oxidative stress, which are related to the development of various blood biochemical-related diseases, such as metabolic syndrome, type 2 diabetes, cardiovascular disease, and liver diseases (Lin et al. 2012; den Besten et al. 2013; Koh et al. 2016; Chambers et al. 2018). Currently, there only exists limited research on the regulation of cholesterol, triglycerides, and blood glucose in chickens, especially with regard to multi-type genetic variants and joint regulation via microbiota in multi-gut segments. Therefore, this study conducted host whole-genome sequencing and whole-gut microbiota 16S rRNA sequencing to explore their interactive regulation on blood biochemical indicators in a population of 206 broiler chickens.

Blood biochemical indicators have a close relationship with the growth and production traits of livestock and poultry. Here, we calculated the phenotypic and genetic correlations between five blood biochemical indicators and 19 growth traits. The results showed that both CHOL and LDL-CH were negatively correlated with growth traits such as body weight, abdominal fat content, and muscle content, regardless of phenotype or genetic correlation. This indicates that an increase in the levels of these blood biochemical indicators may lead to a decrease in growth traits. Previous studies have also suggested that weight loss is commonly associated with elevated HDL-CH levels (Ginsberg 2000; Singh et al. 2007). This may be due to the augmented oxidation of fatty acid and energy expenditure that results from high cholesterol levels, ultimately leading to a decrease in body fat. In addition, glucose is also an important energy source for chicken growth, and its concentration affects the growth rate and feed efficiency of chickens (Bandyopadhyay and Das Mohapatra 2009; Hussein et al. 2018). Elevated levels of blood glucose, triglycerides, and cholesterol are associated with impaired reproductive performance and decreased milk production in dairy animals (Karcagi et al. 2010). It should be noted that the relationship between blood biochemical indicators and livestock growth traits may be influenced by various factors such as gender, age, lifestyle, and health status. Therefore, future research should further explore the complex relationships between these factors and establish more accurate models to predict their relationships.

We estimated the heritability of five blood biochemical traits based on whole-genome SNPs. Our study found that the heritability of five blood biochemical traits in chickens is low to moderate (0.15 ~ 0.24). However, there are significant differences in the heritability of blood biochemical indicators among different species, and even within the same species. For example, in humans, cholesterol has a relatively high heritability. Based on family and twin studies, the plasma level of HDL-CH appears to have a strong genetic basis, with heritability estimated at 40–60% (Lusis et al. 2004; Qasim and Rader 2006). In pigs, the heritability of cholesterol is moderate to high, ranging from 0.23 to 0.58 (Manunza et al. 2014). The heritability of serum cholesterol in chickens ranges from 0.14 to 0.57 in different studies (Dong et al. 2015; Zhang et al. 2018).

In order to capture the impact of multiple genetic variations on blood biochemical indicators, we conducted GWAS analysis based on SNPs, indels, and SVs. In GWAS analysis, the relatedness matrix is used to correct the potential confounding effect of population structure and to reduce the false positive rate. We constructed relatedness matrices based on the standardized genotype matrices of SNPs, indels, and SVs respectively, and found the relatedness matrix based on SVs exhibited significant dissimilarities with those obtained from SNPs and indels. The possible reasons include the following: (1) short-read sequencing is limited in its ability to detect large fragments of SVs (Ho et al. 2020). (2) Insufficient sequencing depth—SV detection has much higher requirements for sequencing depth than single base or small fragment variation detection. (3) The limited sample size leads to a reduced number of identified SVs.

We performed SNPs, indels, and SV GWAS analysis on five blood biochemical indicators and found the *DOCK11* and *RAP2C* genes are the common ones detected by both SNP GWAS and indel GWAS for HDL-CH. Additionally, SV GWAS identified two genes, *NTS* and *BOP1* in HDL-CH. *DOCK11* gene functions as a guanine nucleotide exchange factor (GEF) that is dependent on MATK, a cytoplasmic tyrosine kinase (Ide et al. 2022). The *DOCK11* protein promotes the GDP/GTP exchange of CDC42, thereby activating the CDC42 protein and regulating various cellular processes. Studies have shown that CDC42 controls a multi-transmembrane protein-Niemann-Pick C1-like 1 (NPC1L1) to mediate the absorption of dietary and biliary cholesterol through vesicular endocytosis. Furthermore, some studies have found that CDC42 can regulate the phosphorylation state of 3-hydroxy-3-methylglutaryl-CoA reductase (HMGCR), thereby affecting the rate of cholesterol synthesis. Then, for the *RAP2C* gene, it encodes proteins that belong to the Ras superfamily of small GTPases. It has been reported that overexpression of *RAP2C* may increase the phosphorylation level of protein kinase B (Akt), subsequently promoting the activation of mammalian target of rapamycin complex 1 (mTORC1) and leading to the activation of sterol regulatory element-binding protein (SREBP) nuclear translocation and gene transcription, thereby regulating cholesterol biosynthesis (J et al. 2018). *NTS* encodes a common precursor for two peptides, neuromedin N and neurotensin. Neurotensin is a neuropeptide that has been shown to be involved in the regulation of fat metabolism. Studies have demonstrated that *NTS* can affect insulin secretion and the metabolic activity of adipocytes, thereby influencing the overall balance of fat metabolism in the body. In addition, NTS can also regulate the release of neurotransmitters and activate the hypothalamic–pituitary–adrenal axis, further modulating fat metabolism (Feng et al. 2022). *BOP1* is involved in regulating signal transduction through the p53 class mediator pathway, and p53 transcriptionally regulates squalene epoxidase to repress cholesterol synthesis (Sun et al. 2021). In terms of BG, three types of GWAS have identified three candidate genes: *GDPD5*, *DHDH*, and *KCNIP1*. These genes have been shown to be directly or indirectly involved in glucose regulation and are associated with insulin signaling, glucose transport, and insulin secretion. *GDPD5* is a phosphodiesterase gene involved in glucose metabolism and insulin secretion regulation. *DHDH* is a member of the ketone metabolism pathway and is related to insulin signaling and glucose transport. The protein encoded by *KCNIP1* is related to insulin secretion and insulin sensitivity. These findings contribute to a better understanding of the genetic mechanisms underlying the regulation of CHOL and BG and provide potential targets for improving the growth performance and health status of livestock and poultry.

In this study, a higher microbiability (*m*^2^) value indicated a greater influence of the gut microbiota on the host phenotype. For total CHOL, LDL-CH, and HDL-CH, cecal microbiota play more important roles than other gut segments, with *m*^2^ values of 0.20, 0.13, and 0.24, respectively. The cecum is a crucial part of the digestive tract; some bacteria presented in the cecum such as *Fibrobacter*, *Clostridium*, *Odoribacter*, and *Akkermansia muciniphila* can degrade dietary fiber, produce short-chain fatty acids, promote fat oxidation, reduce fat synthesis, and affect cholesterol metabolism (Parada Venegas et al. 2019; Cani et al. 2022). For TG and BG, the contribution of the jejunal microbiota was found to be the largest. The microbiota in the jejunum can also degrade complex carbohydrates and produce short-chain fatty acids, such as propionic, butyric, and acetic acid. Certain jejunal microbiota, such as *Lactobacillus*, *Saccharomyces boulardii*, and *Butyricicoccus pullicaecorum*, play an important role in glucose metabolism (Chambers et al. 2018). Moreover, certain species of *Firmicutes* have been shown to improve insulin sensitivity, reduce fat accumulation, and lower blood lipid levels by regulating the level of GLP-1 in the gut, thus preventing metabolic diseases such as diabetes and obesity.

The correlation and two-tailed analysis revealed an intricate connection between *RF39* and *Clostridia_UCG.014* with CHOL and TG. *RF39* is a family within the order *RF39* of the phylum *Bacilli*. Research on *RF39* is still relatively limited, but some studies suggest that *RF39* is associated with human metabolic balance. For example, a clinical study found that the abundance of *RF39* was reduced in patients with type 2 diabetes (Zheng et al. 2021). *Clostridia_UCG.014* is a bacterial taxon belonging to the class *Clostridia* in the phylum *Firmicutes*. Studies have shown that a decrease in the abundance of *Clostridia_UCG.014* is associated with obesity (Li et al. 2021). In addition, a study has reported a positive correlation between *Clostridia_UCG-014* and blood glucose levels (He et al. 2022). *Streptococcaceae* is a Gram-positive coccus family that includes various pathogenic bacteria such as *Streptococcus pyogenes* and *Streptococcus pneumoniae* (Wilkening and Federle 2017). However, the *Streptococcus* and *Lactococcus* genera under this family are important bacteria in industrial production that can produce lactic acid through fermentation (Kolling et al. 2012; Sahoo and Jayaraman 2019). Currently, there is limited research on the role of *Streptococcaceae* in human or livestock metabolism.

In order to investigate the potential relationship between host genetics and gut microbiota, we performed SNPs, indels, and SV mGWAS analysis on all families in each gut segment and found that the presence of different microbes in different gut segments is regulated by multiple types of genetic variations and the interaction between host genetics and gut microbiota. Among these families with significant loci, *Staphylococcaceae* in the ileum and *Veillonellaceae* and *Neisseriaceae* in the jejunum were found to be correlated with blood biochemical indicators. *Staphylococcaceae* is a common family of spherical bacteria, among which the only genus detected in our study, *Staphylococcus*, participates in important metabolic reactions such as lactate fermentation and redox reactions. Literature suggests that the abundance of *Staphylococcus* is positively correlated with fasting blood glucose levels, as well as 1-h and 2-h postprandial blood glucose levels (Hu et al. 2021). The key candidate genes of this family, *LDAH* and *APOB*, are important cholesterol-regulating genes. The *LDAH* gene encodes L-lactate dehydrogenase A-like protein, which is an NAD(H)-dependent enzyme. Studies indicate that *LDAH* is associated with changes in LDL cholesterol (Kory et al. 2017). The *APOB* gene encodes the major carrier protein, apolipoprotein B-100, of low-density lipoprotein (LDL). Apolipoprotein B-100 is the only carrier protein on LDL particles and transports cholesterol and other lipid molecules in the body (Kory et al. 2017). Our study suggests that *Staphylococcaceae*, *LDAH*, and *APOB* genes may interact in cholesterol utilization. Additionally, *Veillonellaceae* is capable of producing succinate, and an elevated level of circulating succinate is associated with impaired glucose metabolism (Serena et al. 2018). This family is associated with an important candidate gene, *TOX2*. *TOX2* is expressed in pancreatic islet cells and is involved in regulating the synthesis and secretion of insulin, thus affecting blood glucose levels (Azarova et al. 2021). Our study suggests that the *TOX2* gene may interact with *Veillonellaceae* in blood glucose metabolism. *Neisseriaceae* is a family of Gram-negative bacteria, including many bacteria that can cause human diseases, such as *Neisseria gonorrhoeae* and *Neisseria meningitidis*. This family has candidate genes such as *EHBP1*, *CEP68*, *COPS9*, and *EED*. *EHBP1* (EH domain-binding protein 1) is a gene that encodes a protein involved in intracellular signaling and membrane trafficking processes. *EHBP1* has been implicated in various cellular functions, including the regulation of membrane receptor signaling, the formation of membrane-bound vesicles, and the control of cell migration (Rai et al. 2020). *CEP68*, also known as centrosomal protein 68, is a gene that encodes a protein involved in cellular processes related to the centrosome. *CEP68* is of interest in cell biology and genetics research, as its dysfunction or mutations can lead to centrosome-related abnormalities, which are associated with certain developmental disorders and diseases (Perkins et al. 2019). *COPS9* is a gene that encodes a subunit of the *COP9* signalosome complex. This complex is involved in the regulation of protein degradation and is associated with the ubiquitin–proteasome system (Füzesi-Levi et al. 2020). *EED* is a gene that encodes a protein known as Embryonic Ectoderm Development. *EED* is a component of the Polycomb Repressive Complex 2, which plays a crucial role in gene regulation (Cook et al. 2021). However, further research is needed to explore their interactions with *Neisseriaceae*.

Our study has found that blood biochemical indicators are regulated by both host genetics and gut microbiota. Multiple candidate genes were identified for the regulation of CHOL, HDL-CH, and BG. Notably, *DOCK11* and *RAP2C* were detected in both SNPs and indels-GWAS for HDL-CH. The cecal microbiota exerted the greatest influence on cholesterol levels, whereas the jejunal microbiota had the most substantial effect on TG and BG levels. Additionally, we identified two families, *RF39* and *Clostridia_UCG.014* in the cecum, significantly correlated with CHOL. In addition, we also found that the presence or absence of certain families is genetically regulated and that some of these microbiotas interact with host candidate genes to regulate blood biochemical indicators, including *Veillonellaceae*, *Neisseriaceae*, and *Staphylococcaceae*. Our results provide novel insights into the contribution of the host genetics and gut microbiota to chicken blood biochemical indicators and may aid in developing strategies to manipulate blood biochemical indicators and their associated growth traits.

### Supplementary Information

Below is the link to the electronic supplementary material.

Supplementary file1 (PDF 1539 KB)

Supplementary file2 (XLSX 132 KB)

## Data Availability

The raw data are available from the Sequence Read Archive with accession numbers PRJNA449436, PRJNA449437, and PRJNA449438.
