# Supplementary material for: Host genetics and gut microbiota jointly regulate blood biochemical indicators in chickens
Source: Appl Microbiol Biotechnol. 2023 Oct 4;107(24):7601–20. doi: 10.1007/s00253-023-12814-8 (PMC10656342; doi:10.1007/s00253-023-12814-8)
Supplement: Supplementary file 1 — Supplementary file1 (PDF 1539 KB) [file 253_2023_12814_MOESM1_ESM.pdf]

Supplementary Figures

Journal name: Applied Microbiology and Biotechnology

Manuscript Title: Host genetics and gut microbiota jointly regulate blood biochemical indicators in chickens

The names of the authors: Xinwei Jiang[1]; Boxuan Zhang[1]; Fangren Lan[1]; Conghao Zhong[1]; Jiaming Jin[1]; Xiaochang Li[1]; Qianqian Zhou[1]; Junying Li[1]; Ning Yang[1]; Chaoliang Wen[1*]; Congjiao Sun[1*]

[1]Department of Animal Genetics and Breeding, College of Animal Science and Technology, China Agricultural University, Beijing 100193, China

[*]To whom correspondence should be addressed. Tel: +861062734885; Email: cjsun@cau.edu.cn; Correspondence may also be addressed to Chaoliang Wen. Tel: +8618810642461; Email: clwen@cau.edu.cn.

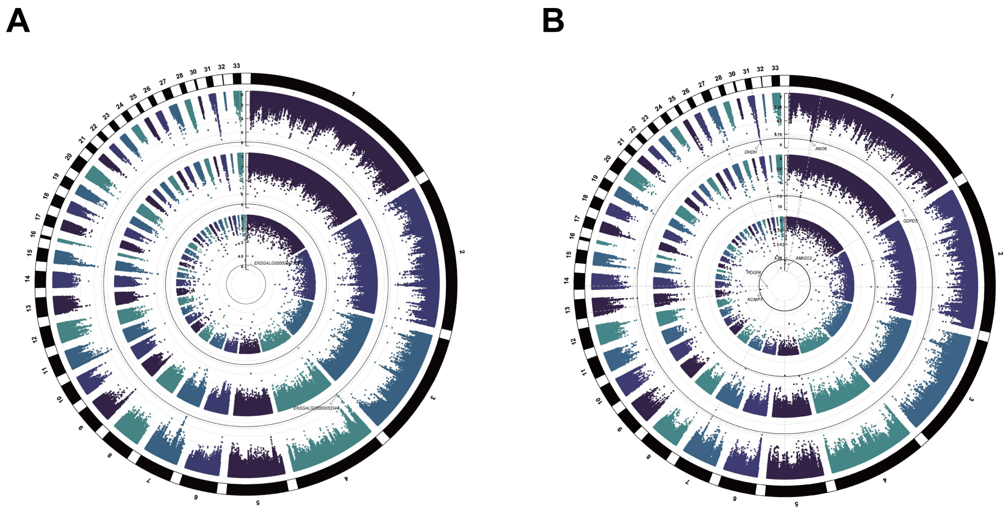

Supplementary Fig. S1. Circular-Manhattan plot of CHOL and BG. (**A**) The Circular-Manhattan plot of CHOL. From the outer circle to the inner circle are the SNPs, Indels and SVs GWAS. The horizontal black solid and grey dashed lines indicate genome-wide significance and suggestive significance thresholds (for SNPs, significant and suggestive significant thresholds were 3.26 and 6.51; for Indel they were 3.76 and 7.51; for SVs the significant threshold was 5.42). (**B**) The Circular-Manhattan plot of BG.

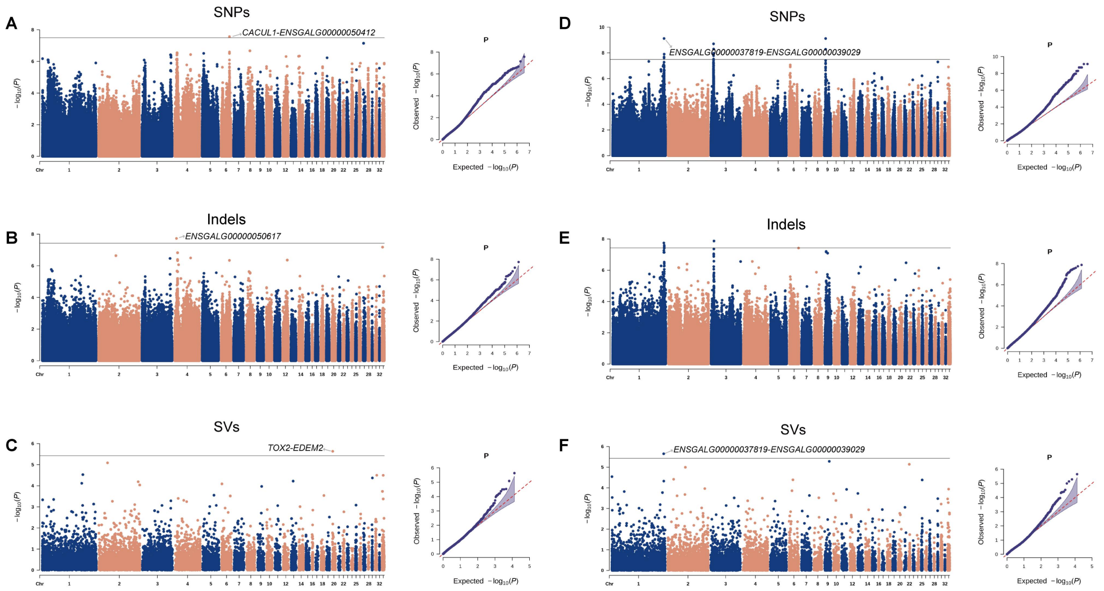

Supplementary Fig. S2. mGWAS results of jejunal *Veillonellaceae* and *Neisseriaceae*. (**A-C**) are the Manhattan and QQ plots of the SNPs, indels and SVs mGWAS results of jejunal *Veillonellaceae.* (**D-F**) are the Manhattan and QQ plots of the SNPs, indels and SVs mGWAS results of jejunal *Neisseriaceae.* The horizontal black lines indicate significance thresholds.
